# The Impact of Blame Attribution on Moral Contagion in Controversial Events

**DOI:** 10.3390/e27101052

**Published:** 2025-10-10

**Authors:** Hua Li, Qifang Wang, Renmeng Cao

**Affiliations:** 1 School of Journalism and Communication, Beijing Normal University, Beijing 100875, China; lihua@bnu.edu.cn (H.L.); fangclouds@mail.bnu.edu.cn (Q.W.); 2 Center for Computational Communication Research, Beijing Normal University, Zhuhai 519087, China

**Keywords:** attribution theory, moral-emotional language, information diffusion, issue context, user identity, Weibo

## Abstract

Controversial events are social incidents that trigger wide discussion and strong emotions, often touching on public interests, moral judgment, or social values. Their diffusion typically involves moral evaluations and affect-laden language. Prior work has mostly examined how the quantity of moral and emotional words shapes diffusion, while largely overlooking blame attribution—that is, whether audiences locate the cause of a controversial event in individual actions or in social structures, across different contexts. Using 189,872 original Weibo posts covering 105 events in three domains— street-level bureaucracy (SLB; individual attribution), education governance (EG; structural attribution), and gender-based violence (GBV; mixed attribution)—we estimate negative binomial models with an interaction between word type and account verification and report incidence rate ratios (IRR). Moral contagion is strongest for SLB (IRR = 1.337) and attenuated for EG (IRR = 1.037). For GBV, moral-emotional language decreases reposts (IRR = 0.844). Unverified accounts amplify the diffusion advantage of moral-emotional wording for both individually and structurally attributed issues, with the largest gains in SLB. When disaggregating by valence and discrete emotions, fear-type moral-emotional words are positively associated with reposts in GBV (IRR = 1.314). Theoretically, we shift the question from whether moral contagion occurs to when it operates, highlighting attribution tendencies and verification status as key moderators. Empirically, we provide cross-issue evidence from large-scale Chinese social media. Methodologically, we offer a replicable workflow that combines length-normalized lexical measures with negative binomial models, including interaction terms.

## 1. Introduction

Public evaluations of controversial events on social media commonly express moral stances and emotional bias. Language that blends moral judgment with emotion can attract attention and influence public opinion [1,2]. Yet the same cue does not travel equally well across all issues [3]. Weibo is a Chinese social platform similar in functionality to Twitter. In settings like Weibo, where institutional actors and ordinary users interact under invisible moderation, how people assign blame—to individuals or to systems—can change diffusion dynamics and, by extension, the tone of public life [4,5]. These dynamics matter because they can amplify polarization in events that focus on individual deviant behavior while muting concern for system-level failures.

Prior work shows that moral-emotional wording can boost online diffusion [6], but effects are different across domains [3]. In events focusing on individual behavior, narratives highlight the actions of specific people, inviting individual blame; in governance disputes, coverage foregrounds abstract rules and institutions, inviting structural attributions [7] (p. 482). These contrasts should have an influence on how moral-emotional language performs. In the Weibo context, media accounts post more cautiously, whereas unverified users rely on affective appeals to spur peer reposting. This pattern is consistent with professional media’s credibility norms and with the platform’s reposting incentives [4,8].

Despite rich theorizing, we lack comparative, cross-issue evidence from non-Western platforms that identifies when moral contagion strengthens, weakens, or reverses. We also know little about how user identity moderates these effects in practice. Without such evidence, people may overgeneralize from highly polarizing cases, and platforms may unintentionally give more visibility to incendiary content through their curation. Our results preview the stakes: moral-emotional language is far more impactful in SLB than in EG, while in GBV, fear-type moral-emotional language drives diffusion; moreover, unverified users benefit most from affect-heavy wording, while verified accounts see muted or even negative returns. Understanding these contingencies is essential for responsible amplification and for designing guardrails that do not simply reward the loudest voices.

This study asks when moral-emotional language increases sharing and for whom. We focus on attribution framing—whether audiences read a problem as caused by individual misconduct or by structural conditions—and on user identity (verified vs. unverified) [9] (p. 16). Our goal is to identify the boundary conditions of “moral contagion” on social media and to translate them into actionable guidance for communicators and platforms. Concretely, we compare three issue contexts that naturally differ in attribution: street-level bureaucracy (SLB, individual blame) [10], education governance (EG, structural blame) [11], and gender-based violence (GBV, mixed attribution) [12,13], and test whether moral emotional language is affected by topic attributes and user identity.

To this end, we test a model of moral contagion that centers attribution framing and user identity, asking when moral-emotional language boosts reposts and when it fails to do so.

Our contributions are fourfold. (1) We describe the contextual boundary conditions of moral contagion, showing it is conditional rather than universal—robust in individual-attribution settings (SLB), attenuated or null in structural frames (EG), and reversed in mixed contexts (GBV) where fear-type moral-emotional language predominates. (2) We demonstrate that user identity influences the effect of moral contagion: unverified accounts capture the largest gains from moral-emotional language, whereas verified or institutional actors do not. (3) Methodologically, we introduce a transparent, replicable workflow that integrates length-normalized linguistic measures with topic classification and moderation analysis, tailored to short-text platforms. (4) We distill practical implications for platform design and public communication under Chinese platform norms. Taken together, these contributions shift the research agenda from asking whether moral contagion exists to specifying when it occurs—and for whom.

## 2. Literature Review

### 2.1. Contextual Boundaries of Moral-Emotional Diffusion

Moral contagion theory [6] posits that language combining moral and emotional content diffuses more widely than language containing either element alone. The words that intersect the emotional dictionary and the moral dictionary are moral-emotional words. That is to say, when posting comments on controversial events on social platforms, compared with purely moral words and purely emotional words, words that contain both moral values and emotional signals are more likely to promote the forwarding of the post. The theory holds that moral language signals group identity and norms, while emotional language provides the motivation to share. Together, these elements produce a synergistic effect. Analyzing Twitter debates on contentious topics, Brady et al. [6] found that each moral-emotional word in a tweet increased its expected retweet count by approximately 20%.

The Motivation–Attention–Design (MAD) model [1] further explains this amplification. It posits that users are motivated to share moral-emotional content to reinforce group identity. This content naturally captures attention due to its salience, and platform design (e.g., likes, algorithms) amplifies these motivational and attentional biases [14]. This mechanism motivates our first hypothesis:

**H1:**  *Posts containing moral-emotional language will be reposted more than posts containing only moral or only emotional language.*

However, scholars have recently questioned whether “moral contagion” generalizes across topics and contexts [3]. Much of the strongest evidence to date comes from polarized issues (e.g., gun control) on Western platforms [6], where what counts as a “polarizing topic” can be subjective, and effect sizes vary by domain. In some polarizing topics, moral-emotional words even predict reduced sharing, indicating that moral contagion does not apply universally [3]. These results do not undermine moral contagion; they point to its boundary conditions. Guided by the MAD framework [1], we treat user sharing motivations, context-driven selective attention, and platform design as the key boundary conditions of moral contagion. Our study examines these conditions in a non-Western setting and offers an exploratory test of when moral-emotional language amplifies—and when it fails to amplify—sharing.

China’s content-moderation system permits some criticism but seeks to deter large-scale collective expression [4]; this institutional context partly shapes how discussions of controversial events spread on Chinese social media. In this moderated space, institutional and official accounts play an outsized agenda-setting role and frequently function as opinion hubs [5,15]. Because verified accounts generally reach larger audiences, they tend to show higher baseline diffusion in routine contexts [16]. However, audiences often read low-arousal or neutral language as more credible [8]. For verified professional or institutional accounts, using moral-emotional language can therefore backfire—prompting doubts about credibility and reducing willingness to repost, especially on controversial events. By contrast, for unverified users, moral-emotional language can signal in-group alignment and mobilize like-minded audiences [6], consistent with the echo-chamber dynamics observed on Weibo [17]. Additionally, posts by conservative-leaning users that employ moral-emotional language are more likely to spread [18], suggesting that moral contagion resonates more readily with broad, non-elite audiences. In such cases, emotionally charged moral claims from grassroots accounts can diffuse rapidly on Weibo, at least prior to the full salience of any collective-action risks.

We therefore hypothesize the following:

**H2:**  *For posts containing moral-emotional language, unverified user status will predict more reposts than verified user status.*

### 2.2. Arousal-Driven Mechanisms of Emotional Sharing

Emotional contagion is a fundamental mechanism of social cohesion [19]. Emotional tweets are shared more often and faster than neutral ones [20], and emotional interaction can become part of a society’s social beliefs and reshape relational structures, thereby shaping how we see and judge social problems [2].

Finer-grained evidence shows that emotional valence does not have a uniform impact across settings. In news, positive content tends to spread more virally [21]; in rumor diffusion, false rumors go viral when they contain more positive-emotion words [22]. By event type, positive emotion builds up for highly-anticipated events, whereas unexpected events are marked by negative emotion; correspondingly, positive information reaches broader audiences, while negative information spreads faster [23]. In political contexts, negative affect more reliably boosts sharing [24]. Together, these patterns indicate that the effects of emotional valence are context-dependent rather than universal.

Compared with valence, physiological arousal provides a clearer framework for explaining why different emotions spread to different degrees. High-arousal states—positive (e.g., awe) or negative (e.g., anger, anxiety)—promote viral diffusion, whereas low-arousal emotions (e.g., sadness) suppress it [21]. Studies of discrete emotions further show that anger is more contagious than joy, an effect amplified by weak social ties [25]. This perspective motivates our first research question:

**RQ1:**  *Across different valences and categories, which specific moral-emotional language best predicts reposts?*

### 2.3. Attribution Frames and Issue Types

According to attribution theory, different emotional responses are evoked depending on where blame is placed. When individuals are held responsible (internal attribution), the common emotional reaction is anger [26]. When the cause is seen as structural (external attribution), the response is more likely to be sympathy [26].

“Attribution in terms of impersonal and personal causes, and with the latter, in terms of intent, are everyday occurrences that determine much of our understanding of and reaction to our surroundings” [9] (p. 16). When observing others, people tend to underestimate situational factors and overestimate dispositional ones. This cognitive bias, known as the fundamental attribution error, is a robust phenomenon observed even in trained psychologists [27] and is likely widespread in the general public.

Attributing social problems is inherently political. While the sociological imagination encourages linking individual biographies to broader social structures [28] (pp. 10–11), the dominant tendency is often to “blame the victim” [29] (pp. 11, 17). This ideology individualizes systemic issues, framing them as personal failings that require exceptional, case-by-case solutions rather than universal reforms. This focus on personal deficits over structural causes ultimately reinforces the existing social order [29] (p. 19).

Media narratives shape public attribution by framing the causes of social problems [7] (pp. 477, 493). News coverage that focuses on individual actions and choices encourages internal attributions. Conversely, coverage that examines systemic or social causes promotes external, situational attributions [7] (p. 482). Therefore, person-centric event narratives are more likely to lead to individual blame, while abstract, institution-focused narratives are more likely to lead to structural blame.

To operationalize these framing effects in our study, we next define three issue categories that map media narratives to attributional logics: attributable to individual actions, attributable to social structures, and attributable to a mix of the two.

- Attributable to individual actions: Events framed mainly as the choices, intentions, or misconduct of specific people (e.g., a police officer, a teacher, a local official). The problem is treated as a discrete event caused by identifiable actors, and solutions focus on disciplining, rewarding, or replacing those individuals.
- Attributable to social structures: Events framed as the result of rules, incentives, institutions, or broad social conditions (e.g., laws, hiring systems, cultural norms). The problem is treated as systemic and persistent across cases, and solutions emphasize policy or organizational reform.
- Attributable to a mix of individual actions and social structures: Events where narratives link specific actors’ behaviors to the larger systems that enable or constrain them. Both personal agency and structural conditions are presented as necessary parts of the explanation, and solutions combine accountability for individuals with reforms to rules or contexts.

We compare three types of issues that naturally encourage different attributions. For individual attribution, we selected street-level bureaucracy, which involves the concrete actions of specific people. We then selected education governance as a case of structural attribution, as it involves abstract institutions. To further examine how issues that combine individual and structural attributions unfold, we analyze gender-based violence as a mixed case. By analyzing the moral-emotional content and repost counts of posts within these domains, we can examine the boundary conditions of the theory.

The concept of street-level bureaucracy, introduced by Lipsky [10,30], refers to frontline public service personnel who exercise discretion under conditions of limited resources and ambiguous goals, effectively shaping how policies are implemented. In China, such cases often involve urban management officers or police. Media narratives of these incidents usually highlight the actions of individual officials (e.g., “a police officer kicked a student”), which encourages the public to attribute the problem to personal misconduct rather than broader structural factors.

Education governance refers to controversies over the structural arrangements, rules, and policies within the education system. In contrast to the concrete actions of SLB, EG is an abstract, multi-level system where actors are often faceless institutions [11]. Media coverage of EG issues therefore focuses on macro-level rules, guiding the public toward situational or structural attributions.

Although Weibo shares the core microblogging affordances of Twitter/X, the cultural and institutional environment in which those affordances operate is distinct. While platforms like Weibo possess an open structure capable of empowering users to spread information publicly [31], the same moral-emotional cue can have weaker or stronger effects depending on whether the public reads a problem as structural (stable, systemic) or personal (discrete, individual-level). Structural frames implicitly raise the perceived potential for wide-scale collective expression—making such content more likely to be constrained under moderation rules [4]. —whereas person-centric incidents pose less threat to institutional stability and thus allow moral-emotional content to travel farther.

We therefore predict the following:

**H3:**  *Moral-emotional language will have a weaker effect on reposts in the context of education governance compared to street-level bureaucracy.*

Then, we follow the United Nations High Commissioner for Refugees (UNHCR) [12] and World Health Organization (WHO) [13] to define gender-based violence as violence, discrimination, or coercion directed at individuals—especially women and gender minorities—on the basis of gender, gender identity, or socially constructed gender roles. Manifestations include physical and sexual violence, verbal abuse and harassment, humiliating or stigmatizing speech (including online), and institutional or systemic inequities; these harms can occur in both private and public settings and carry severe, sometimes lifelong, consequences.

“Violence, Peace and Peace Research” [32] distinguishes personal (direct) from structural (indirect, system-level) violence and argues they are intertwined rather than separable: individual actions are patterned by institutional arrangements and social norms. Building on this, ecological models of violence against women conceptualize GBV as arising from the interaction across levels—individual traits and relationships (micro), situational and organizational contexts (meso), and cultural, legal, and economic structures (macro) [33]. Taken together, these frameworks justify treating GBV as a mixed attribution domain in which personal agency and structural conditions jointly shape expression and diffusion.

## 3. Materials and Methods

### 3.1. Data Collection and Issue Classification

Our data is drawn from the Sina Weibo Online Emergency Public Opinion Dissemination Dataset provided by the 2025 Micro Hotspot Big Data Research Institute, which contains 349 trending events from 2024 onward. The provider clustered Weibo posts to events by lexical similarity and assigned unique IDs to all events. We then collected the original Weibo posts for these events, extracting the full text, user verification status, follower count, media type and repost count for analysis.

We employed a human–Artificial Intelligence (AI) collaborative approach for issue classification, leveraging the ability of Large Language Models (LLMs) to replicate expert annotations while maintaining human supervision for accuracy [34,35]. Our process adapts the workflow from Chew et al. [36] to classify the event titles.

From the universe, we drew two random subsets for calibration. In each subset, two trained coders independently labeled the event titles into four mutually exclusive categories— street-level bureaucracy (SLB), education governance (EG), gender-based violence (GBV), and other—using short operational definitions. We then prompted Gemini 2.5 Pro to code the same events (full prompt in Appendix C.2). Human labels and model outputs were compared; both calibration rounds showed high agreement, leading us to proceed with model-assisted coding of the full corpus while keeping human oversight for flagged or ambiguous cases. This “calibrate → audit → scale” design follows recent guidance on LLM-assisted deductive coding and on integrating LLMs into workflows that emphasize human oversight and triangulation [36,37].

After the two audits confirmed stable agreement, we applied the same instructions and settings to all 349 events and retained human spot-checks for any low-confidence determinations. Following data quality screening, we excluded events with missing critical fields (e.g., user IDs) required for downstream analyses. The resulting research sample comprised 42 SLB events, 46 EG events, and 17 GBV events; the remainder were labeled “other” and not analyzed as focal categories. Our procedure mirrors the recommended final step in LLM-assisted content analysis—using the model to create the final coded dataset once non-inferiority to human coding has been demonstrated—while documenting prompts, model name, and run parameters for replicability [36,37] (see Appendix C).

After initial cleaning (de-duplication, removal of missing values, and trimming repost outliers at the 0.1% level), the final analytic samples comprised 23,730 posts for SLB (42 events), 97,731 posts for EG (46 events), and, with the addition of the GBV topic, 68,411 posts for GBV (17 events).

### 3.2. Operationalization of Variables

Our dependent variable is the repost count, a key indicator of information diffusion [6]. Because repost counts are over-dispersed count data (i.e., the variance is much larger than the mean), we selected a statistical model appropriate for this distribution.

Our core independent variables measure the language used in each Weibo post. We constructed these variables using two Chinese lexicons: the Chinese Moral Foundation Dictionary (C-MFD) 2.0 moral lexicon [38] and the Information Retrieval Laboratory of Dalian University of Technology (DUTIR) emotional lexicon [39]. From these, we created three mutually exclusive categories:

- Moral-Emotional Words: Words present in both lexicons (*n* = 1957);
- Distinctly Moral Words: Words unique to the moral lexicon (*n* = 3747);
- Distinctly Emotional Words: Words unique to the affective lexicon (*n* = 25,358).

To control for post length, we normalized the count of each word type using the following formula:

(1) $$\mathrm{LinguisticVariable}=\left(\frac{f}{N+1}\right)\times 100$$

The normalized metric represents the number of words of a given type per 100 characters, which improves the interpretability and stability of the model coefficients. Our analysis also includes the following moderator and control variables:

- User Verification (Moderator): Effects-coded as ordinary user (−1) or verified user (+1);
- Follower Count (Control): To control for user influence, we use the log-transformed number of followers due to the variable’s right-skewed distribution;
- Media Type (Control): Effects-coded as text-only (−1) or multimedia (e.g., images, video) (+1);
- Post Length (Control): The character count of the post is included as an additional control in our robustness checks.

To address RQ1, we created fine-grained sentiment intensity scores for each post. These scores were calculated using a standard Chinese sentiment formula [40] that integrates several components:

- Core Lexicons: The DUTIR lexicon [39] provided word polarity (positive/negative) and discrete emotion categories (e.g., joy, sadness).
- Modifier Lexicons: We incorporated established negation [41] and degree-adverb [42] lexicons.
- Scoring Logic: The formula accounts for a word’s polarity (+1 or −1), its strength (on a 5-point scale), the multiplicative effect of negators, and the weighting of degree adverbs.

This process generated our final exploratory variables: scores for positive/negative emotion, positive/negative moral-emotion, and each discrete moral-emotion category. The formula is as follows:

(2) $$TotalScore=\sum_{i=1}^{n}\left(Intensity_{i}\times Polarity_{i}\times DegreeWeight_{i}\times NegationFlag_{i}\right)$$

### 3.3. Analytic Strategy

We used negative binomial regression to model the repost counts. This method was chosen because our dependent variable is an over-dispersed count, meaning its variance is much larger than its mean. Unlike Poisson regression, which assumes equal mean and variance, the negative binomial model accounts for this over-dispersion and is therefore more appropriate for our data [43].

To test our hypotheses, we modeled repost counts using negative binomial regression. The models included interaction terms to assess the main effects of language type and the moderating effect of user verification. The resulting coefficients are interpreted as incident rate ratios (IRRs):

(3) $$\ln(\mu_{i})=\beta_{0}+\sum_{j=1}^{6}\beta_{j}X_{j,i}+\sum_{k=1}^{3}\gamma_{k}Z_{k,i}$$

where

- *i* is indexes the *i*-th post;
- $\mu_{i}$ is the expected repost count for post *i*;
- $\beta_{0}$ is the intercept;
- *X_j,i_* is the value of the j-th predictor (main effect) for post *i*;
- $\beta_{j}$ is the coefficient for the *j*-th predictor;
- $Z_{k,i}$ is the value of the *k*-th interaction term for post *i*;
- $\gamma_{k}$ is the coefficient for the *k*-th interaction term.

To investigate RQ1, we specified two exploratory models. The first model tests the effect of emotion valence, classifying emotions as positive (joy, good, surprise) or negative (anger, sadness, fear, disgust) based on the DUTIR lexicon while holding all controls constant [39]. Second, we created variables for five discrete moral-emotion categories, excluding two due to low frequency (surprise and anger). In both models, intensity is measured as a normalized frequency per 100 characters. The models are specified as follows:

ln(Reposts) = β_0_ + β_1_⋅Moral Words + β_2_⋅Positive Emotion + β_3_⋅Negative Emotion + β_4_⋅Positive Moral Emotion + β_5_⋅Negative Moral Emotion + β_6_⋅Log Followers + β_7_⋅Auth Type + β_8_⋅Media Type + ε (4)

ln(Reposts) = β_0_ + β_1_⋅Joy + β_2_⋅Good + β_3_⋅Sadness + β_4_⋅Fear + β_5_⋅Disgust (5)

## 4. Results

### 4.1. Main Effects of Language on Reposts and Cross-Issue Differences (Model 1)

Table 1, Table 2 and Table 3 present the negative binomial regression results for the SLB, EG, and GBV topics, respectively.

The results do not support H1 across all issues. In SLB, moral-emotional language has the strongest effect (IRR = 1.337), exceeding distinctly moral (IRR = 1.067) and distinctly emotional (IRR = 1.061) language. In EG, the pattern is similar but smaller: moral-emotional (IRR = 1.037) exceeds distinctly emotional (IRR = 1.022). However, in GBV, the pattern reverses: distinctly emotional language increases sharing (IRR = 1.118), whereas moral-emotional decreases it (IRR = 0.844), and distinctly moral language also decreases it (IRR = 0.952). These findings indicate that moral contagion effects vary by topic and message type.

The data support H3, revealing the context-dependent effectiveness of language strategies. Moral-emotional language was significantly more impactful in SLB (IRR = 1.337) than in EG (IRR = 1.037). These findings suggest that moral contagion is strongest when the media narrative highlights a direct conflict and blames the problem on individual misconduct.

### 4.2. The Moderating Role of User Identity

The analysis supports H2, showing that user verification status acts as a moderator for the diffusion effect of language. Given the complexity of interpreting interaction coefficients directly, we plotted the predicted marginal effects. This graphical analysis demonstrates how the relationship between language and post diffusion differs significantly for verified versus unverified users within each topic.

**Figure 1:**
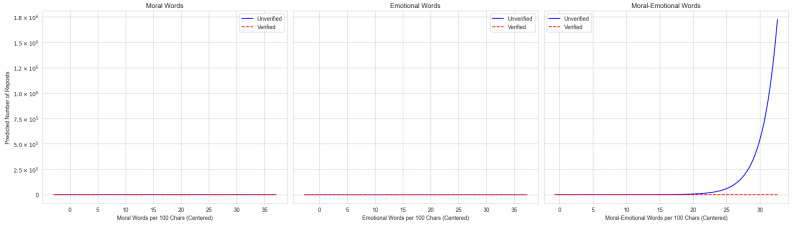
Moderating effect of user authentication on language diffusion in SLB discussions. The plots visualize the predicted marginal effects from the negative binomial regression model. The vertical axis represents the predicted number of reposts, where the label 10^6^ denotes millions (e.g., 1.0 × 10^6^ equals 1,000,000). The steeper upward slope indicates the stronger viral effect of moral-emotional language.

For the SLB topic (Figure 1), a stark divergence emerges between user types. A higher density of moral-emotional language predicts an exponential increase in reposts for unverified users, indicating it can trigger virality. Conversely, this language has no discernible impact on the engagement of verified users, whose repost counts remain low regardless of its use.

**Figure 2:**
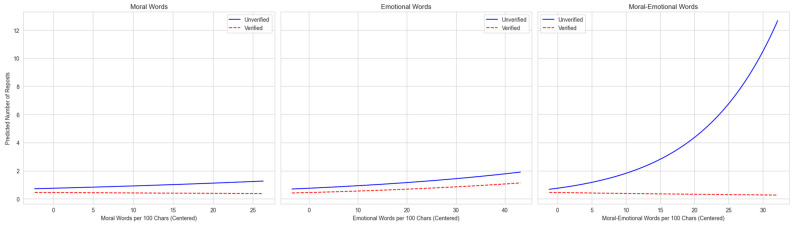
Moderating effect of user authentication on language diffusion in EG discussions. The plots visualize the predicted marginal effects from the negative binomial regression model. The vertical axis represents the predicted number of reposts. The steeper upward slope indicates the stronger viral effect of moral-emotional language.

In the EG topic (Figure 2), the engagement pattern differs. Overall engagement is lower, with predicted reposts for unverified users peaking at approximately 12. For these accounts, the positive relationship between moral-emotional language and reposts is weaker and linear, not exponential.

**Figure 3:**
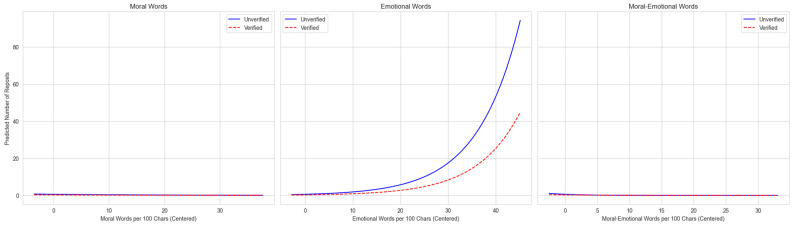
Moderating effect of user authentication on language diffusion in GBV discussions. The plots visualize the predicted marginal effects from the negative binomial regression model. The vertical axis represents the predicted number of reposts. The steeper upward slope indicates the stronger viral effect of emotional language.

For the GBV topic (Figure 3), diffusion is driven primarily by emotional language. As the density of emotional words increases, predicted reposts for unverified accounts rise steeply while verified accounts also gain but plateau at a lower level, indicating that verification attenuates the payoff from emotional language. By contrast, moral-emotional language does not fuel sharing: predicted reposts stay low and even edge downward across the range for both user types, with only minor separation between the lines. Distinctly moral language shows a similarly flat-to-negative pattern near zero.

Taken together, the results show clear moderation by user verification. Unverified users gain most from affective cues: in SLB, moral-emotional language turns viral; in EG, it helps modestly; in GBV, emotion drives the gains. Verified users see little benefit from moral-emotional language and a muted return to emotion in GBV, consistent with credibility-preserving strategies [8]. In short, weaker actors lean on affect to mobilize sharing, while institutional actors keep tone restrained to protect trust.

### 4.3. Robustness Checks

We conducted several robustness checks which confirmed that our core findings are stable. The effect of moral-emotional language and its moderation by user type were unaffected by the following changes:

- Model Simplification: The results held when retaining only moral-emotional words, their interactions, and controls;
- Control Variables: The results were consistent when adding post length or removing media type as controls (except EG);
- Interaction Terms: The results were unchanged when retaining only interactions involving moral-emotional words.

Finally, a bootstrap analysis (1000 resamples) confirmed that the effects are not artifacts of user-level clustering (except EG). Therefore, the findings are robust to alternative model specifications and data structures (see Appendix A for details).

Only the education governance checks raised reliability concerns, likely because this structurally framed topic is disproportionately influenced by users who post multiple times. When the chance of adopting or sharing a message rises with repeated exposure, and cluster size (the number of posts per user) is related to outcomes conditional on covariates, an “informative cluster size” (ICS) problem can arise.

In our data, about 27% of authors contributed more than one post, introducing non-independence. As Table 4 shows, most of these multi-post authors contributed exactly two posts, while authors with five or more posts were rare. Cluster sizes were also highly heterogeneous across topics (ranges in Table 4): SLB: 1–76; EG: 1–380; GBV: 1–466.

**Figure 4:**
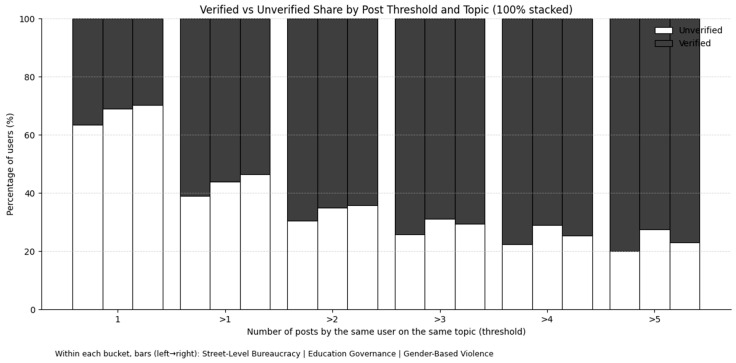
Verified vs. unverified share by post threshold and topic (100% stacked). Each *x*-axis group shows the number of posts by the same user on the same topic (1, >1, >2, >3, >4, >5). Within each group, bars (left→right) correspond to SLB, EG, and GBV. The verified proportion rises steadily as the posting threshold increases (roughly from ~30–40% at one post to ~70–80% beyond >4), indicating that high-activity users are disproportionately verified and the unverified share correspondingly declines.

Moreover, within each topic the share of verified accounts increases as the number of posts per author rises, indicating that highly active users are disproportionately verified. Compositional differences across user types likely account for the observed interaction (see Figure 4).

**Figure 5:**
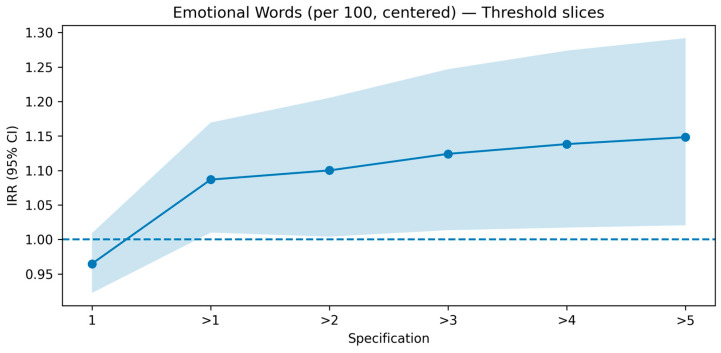
Emotional words—threshold slices. Negative-binomial IRRs with 95% CIs are estimated on six nested subsets by re-introducing multi-post users (1, >1, >2, >3, >4, >5). The IRR shifts from ~0.97 at the single-post slice to ~1.09 at “>1” and then increases slightly as higher-activity users are included, with widening uncertainty bands. This pattern indicates that the apparent positive effect of emotional language in EG is largely driven by highly active accounts.

**Figure 6:**
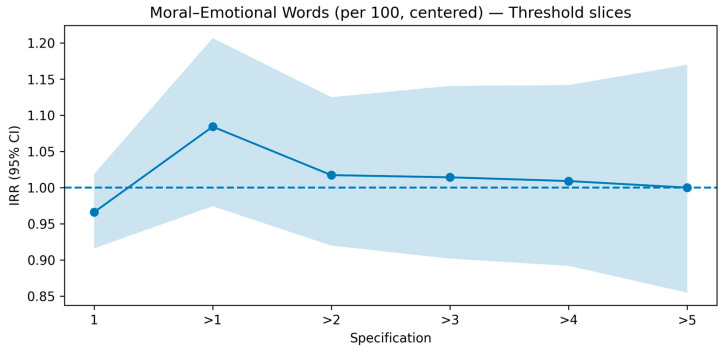
Moral-emotional words—threshold slices. Negative-binomial IRRs with 95% CIs estimated on six nested subsets (1, >1, >2, >3, >4, >5). The IRR rises from ~0.97 at the single-post slice to ~1.08 at “>1”, then tapers back toward ~1.00 as increasingly active users are included, with widening uncertainty. This pattern indicates that the apparent positive effect is largely driven by highly active accounts.

**Figure A2:**
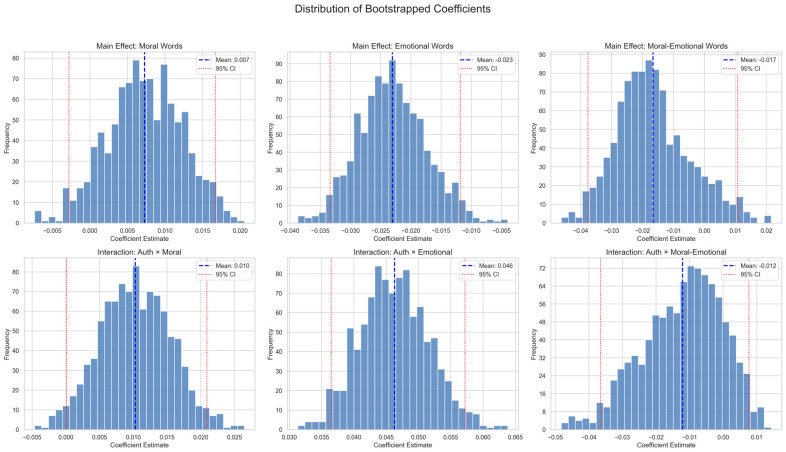
Distribution of bootstrapped coefficients for the EG model. Each panel shows the sampling distribution of a coefficient with the mean (blue dashed) and 95% CI (red). When we mute high-activity users (one post per multi-post user per resample), the emotional and moral-emotional main effects shift toward or below zero and their CIs often span zero, in contrast to the main model. By comparison, moral is near zero/slightly positive, Auth × Emotional is positive, and Auth × Moral-Emotional is negative. Taken together, the bootstrap indicates that the apparent positive effects in EG are sensitive to ICS and largely driven by a small set of highly active accounts.

We conducted two checks on the education governance topic. First, a cluster-aware bootstrap (1000 resamples; one post drawn per multi-post user in each resample) showed that, when the influence of highly active users is muted, the effects of distinctly emotional and moral-emotional language on reposts become small and often trend below unity, indicating weak or even negative associations (see Appendix A.5 Figure A2). Second, we implemented a threshold-slices analysis that incrementally re-introduces multi-post users by estimating separate negative-binomial models on six nested subsets: users with exactly one post (=1), and users with ≥2, ≥3, ≥4, ≥5, and ≥6 posts (notated “>1” through “>5”). The resulting IRR trajectories (Figures “Emotional Words—Threshold slices” and “Moral-Emotional Words—Threshold slices”) reveal a clear pattern: for distinctly emotional language, the IRR crosses the 1.00 line precisely when moving from the single-post slice to “>1” (≈0.97 → ≈1.09) and then creeps upward with wider CIs as more high-activity users are included (see Figure 5); for moral-emotional language, the IRR similarly jumps above 1.00 at “>1” (≈0.97 → ≈1.08) but then flattens back toward ≈1.00 as thresholds increase, with broadening uncertainty bands (see Figure 6). Taken together, these visuals show that the apparent promotive effects in the EG main model are largely induced by a small set of highly active accounts; once we control for this ICS bias, the effects attenuate or reverse.

### 4.4. Exploratory Analysis: Effects of Emotion Valence and Categories

Negative emotions show a diffusion advantage. In SLB (IRR = 1.028) and EG (IRR = 1.03) topics, posts containing negative emotional language were significantly more likely to be reposted, whereas those with positive emotional language were not. But in the GBV topic, positive emotional language reliably increases reposts (IRR = 1.041), whereas negative emotion is not significant.

The effects of polarity are more complex for moral-emotional language. In the SLB topic, both positive (IRR = 1.084) and negative (IRR = 1.055) moral-emotional language significantly promoted reposts. In the EG topic, however, only negative moral-emotional language had an effect of suppressing reposts (IRR = 0.988). Similarly, in the GBV topic, negative moral-emotional language slightly reduces reposts (IRR = 0.993).

Effects differ across discrete emotion categories. An analysis of specific categories (restricted to good, sadness, fear, disgust, and joy due to data sparsity) revealed further distinctions. Moral-emotional language related to good promoted reposts across the SLB topic (IRR = 1.022), while language related to disgust significantly suppressed them in the EG topic (IRR = 0.988). But in the GBV topic, discrete ME categories reveal sharper contrasts: While good-related moral-emotional language is associated with a modest increase in reposts (IRR = 1.069), fear-type moral-emotional words shows a large promotive effect (IRR = 1.314).

These results suggest that future research could benefit from using finer-grained emotional taxonomies to capture how specific emotions influence diffusion (see Appendix B for details).

## 5. Discussion

### 5.1. Summary of Findings

Our exploratory attribution analysis shows that moral contagion is not uniform across issues. When problems are framed at the individual level (SLB), moral-emotional language strongly boosts diffusion; when framed as structural (EG), the effect weakens or reverses once we address high-activity users and cluster bias. In the mixed case (GBV), purely emotional cues increase sharing while moral-emotional cues reduce it, echoing evidence from #MeToo contexts that moralized appeals can backfire in mixed-attribution debates [3]. These patterns suggest that attribution—not emotion alone—conditions when moral language spreads.

User type also matters. Unverified accounts gain most from affect-heavy messaging, consistent with discursive empowerment of non-elite actors. Verified and institutional accounts see muted returns, likely because credibility norms favor a neutral tone [8] and because platform dynamics around public-affairs topics penalize overt moralization [4]. Together, these results point to distinct audience–disseminator fit.

Emotion structure further clarifies the boundary conditions. In GBV, both fear-related and good-related moral-emotional language are associated with increased reposting, whereas sadness-related language is not. High-arousal signals travel; low-arousal signals do not. The implication is both practical and theoretical: amplifying high-arousal moral language can heighten visibility through diffusion, but it may intensify polarization and reduce space for reasoned debate.

These findings advance a conditional view of moral contagion: attribution framing, user type, and emotion structure jointly determine diffusion. We show strong effects for individual-level issues, attenuation or reversal for structural issues, and fear-type moral-emotional language driven diffusion in GBV. We therefore shift the question from “does moral contagion exist?” to “when does it occur?”.

### 5.2. Theoretical and Methodological Implications

This study advances moral-contagion theory on three fronts. First, using large-scale Weibo data, we show that moral contagion is not confined to a single cultural context—consistent with cross-lingual evidence that emotion–virality relationships generalize across languages when modeled via valence, arousal, and dominance (VAD) [44]. Its impact varies by topic and disseminator identity, supporting a contextual, rather than universal, model. Second, by linking contagion to arousal theory, we clarify mechanism: in contentious debates, high-arousal cues—especially negative ones—do most of the work. Arousal, not valence alone, appears to translate moralized content into sharing [21].

Our findings also suggest a social-psychological bridge between attribution and polarization. When issues are framed at the individual level, audiences can more easily locate a responsible agent, often outside their in-group [45]. Prior work shows that perceived out-group hostility is over-detected online and that such perceptions spur reposting [46]. This points to a testable hypothesis: individual-level attribution may heighten out-group antagonism, which in turn amplifies moral contagion. If supported, interventions could target early attribution framing (e.g., encouraging structural explanations) to slow polarization.

Methodologically, we contribute three tools for computational text analysis. We introduce a relative density metric that normalizes word use by text length, improving sensitivity for short posts. We develop a human–AI coding pipeline that combines researcher rules with LLM validation to classify large volumes of social media data at low cost. Following prior work on Chinese sentiment scoring [40], which integrates polarity, intensity, negation, and degree adverbs, we repurpose that formula to analyze moral-emotional valence and discrete emotions in Chinese moral contagion.

Finally, we provide a replicable framework for studying moral and emotional discourse in Chinese social media: custom lexicons, a transparent human–AI workflow, and statistical models that handle clustering and heterogeneity. The framework is portable and can be adapted across platforms and contexts to test when individual-, structural-, or mixed-attribution amplifies or attenuates moral contagion.

### 5.3. Practical Implications

Our findings that moral-emotional language generally boosts diffusion on Weibo, but with sizable heterogeneity by issue domain and user identity (e.g., ≈33.7% in SLB vs. ≈3.7% in EG; identity moderation strongest for unverified users), align with and extend platform-agnostic theories of affective amplification.

This pattern resonates with evidence from Facebook that highly engaged participation within like-minded communities can tilt collective sentiment toward negativity and shape group dynamics—an “echo-chamber” mechanism that helps explain why strong moralized emotion travels farther in conflict-laden, individually attributable topics (SLB) than in structurally framed governance discussions (EG) [47].

At the same time, our topic- and identity-specific contingencies complement recent modeling work that tracks user and community sentiments over time and introduces “cross-contamination” between communities and their neighborhoods; our results suggest that such community-level sentiment dynamics are likely to differ by issue attribution and actor status on Weibo, and they motivate future analyses that couple our diffusion estimates with network-based sentiment evolution frameworks [48].

For platform governance, our findings highlight a tension between maximizing user engagement and maintaining social responsibility. Algorithms designed for engagement may unintentionally amplify the most incendiary and emotionally negative content, increasing exposure to polarizing material and potentially worsening social divisions [1]. This suggests a need to integrate principles of social responsibility directly into the design of content curation and distribution algorithms.

For social actors such as government agencies, media, and non-governmental organizations (NGOs), these findings highlight the critical role of language in communication strategy. The results show that while moral-emotional language can maximize a message’s reach, it also risks oversimplifying complex issues and inflaming conflict. This presents a key ethical dilemma: social actors must balance the strategic goal of effective dissemination against the responsibility to foster reasoned public discourse.

### 5.4. Study Limitations and Future Research

First, our operationalization of attribution by issue category relies on theoretically informed—but ultimately conceptual—distinctions rather than a direct, respondent-level measure of how audiences assign blame. Future work should incorporate more fine-grained, quantitative indicators of attribution—for example, employing a BERT-based feature set and human–AI collaborative coding to classify attributions in comments on polarizing events, then comparing moral-contagion effects across attribution types.

Second, our analyses of heterogeneity by user verification status and by moral-emotional polarity/category are exploratory. While we document systematic differences, we do not fully unpack the mechanisms that generate these patterns. Future studies should move beyond descriptive moderation to probe causal pathways.

Finally, we analyze moral contagion on Weibo to compare attribution effects, not to localize the phenomenon to China. Replications across platforms and across cultural/moderation contexts are needed to validate how attribution shapes moral contagion beyond this setting. Taken together, these limitations point to a clear agenda: pair audience-level attribution measures with mechanism-focused designs, and test the theory across diverse platforms and cultural–institutional contexts.

## 6. Conclusions

This study shows that moral contagion is conditional, not universal. Whether moral-emotional language spreads depends on three levers: attribution framing, user identity, and arousal profile. When issues are framed at the individual level (SLB), moral-emotional cues travel widely; for structural governance topics (EG), the effect weakens or can reverse once we account for highly active users and clustering. In mixed-attribution debates (GBV), diffusion is driven by high-arousal fear, while moral-emotional wording can backfire. Unverified users benefit most from affect-heavy language, whereas verified and institutional accounts see muted returns, consistent with credibility norms and public-affairs dynamics [8].

Theoretically, we shift the question from whether moral contagion exists to when it occurs. A conditional model—anchored in attribution and arousal—better explains variation across topics than emotion alone [6]. Methodologically, we provide a replicable toolkit for Chinese social media: a length-normalized density metric, a transparent human–AI coding workflow, and the adoption of an existing affect-scoring formula (polarity, intensity, negation, degree adverbs).

Practically, the findings highlight a tension for platforms and communicators. Engagement-optimized ranking can over-amplify high-arousal, incendiary content; responsible design should account for attribution cues and user type to avoid amplifying polarization. For public agencies, media, and NGOs, moralized appeals mobilize grassroots supporters yet have limited traction with policy audiences and can oversimplify complex problems.

Our evidence is bounded by conceptual attribution categories and exploratory heterogeneity analyses. Future work should measure audience attributions directly and test these mechanisms across platforms and cultural-institutional settings. A more precise, evidence-based account of how moral contagion operates can clarify why polarizing events escalate on social media, how they can be interrupted, and how online signals reshape offline collective beliefs [2].

## Figures and Tables

**Table 1:** Negative binomial regression (DV: reposts)—SLB. Moral, emotional, and especially moral-emotional language is positively associated with reposts (IRR = 1.34 for moral-emotional), net of controls. Interactions show that verification dampens the payoff of moral-emotional language (IRR = 0.86) and slightly reduces the effect of moral words (IRR = 0.97), while it amplifies the effect of emotional words (IRR = 1.05).

	Coefficient	*p* > |z|	IRR	IRR_CI_2.5%	IRR_CI_97.5%
Intercept	−0.267364	0.000000	0.765394	0.705306	0.830601
Moral Words	0.065249	0.000000	1.067424	1.041346	1.094156
Emotional Words	0.059540	0.000020	1.061348	1.032668	1.090825
Moral-Emotional Words	0.290710	0.000000	1.337377	1.260496	1.418948
Log-Followers	0.573866	0.000000	1.775116	1.736695	1.814387
Authentication Type	−0.248834	0.000000	0.779710	0.717619	0.847173
Media Type	0.818284	0.000000	2.266607	2.089126	2.459166
Authentication Type × Moral Words	−0.030681	0.014607	0.969785	0.946196	0.993962
Authentication Type × Emotional Words	0.048961	0.000383	1.050180	1.022182	1.078945
Authentication Type × Moral-Emotional Words	−0.149819	0.000001	0.860864	0.811626	0.913088

Note: All language variables and followers are mean-centered. User type and media type use effects coding. *n* = 23,730.

**Table 2:** Negative binomial regression (DV: reposts)—EG. Emotional and moral-emotional language show small positive associations with reposts (IRR ≈ 1.02–1.04), while moral language is not significant. Interactions indicate that verification slightly amplifies the effect of emotional words (IRR = 1.02), but reduces the effect of moral-emotional language (IRR = 0.95); the interaction with moral words is marginal.

	Coefficient	*p* > |z|	IRR	IRR_CI_2.5%	IRR_CI_97.5%
Intercept	−0.539663	0.000000	0.582945	0.563792	0.602749
Moral Words	0.006391	0.355392	1.006411	0.992863	1.020144
Emotional Words	0.021411	0.002210	1.021642	1.007729	1.035747
Moral-Emotional Words	0.035958	0.002339	1.036612	1.012883	1.060897
Log-Followers	0.390396	0.000000	1.477566	1.461997	1.493300
Authentication Type	−0.259564	0.000000	0.771388	0.736167	0.808295
Media Type	1.111927	0.000000	3.040211	2.940554	3.143246
Authentication Type × Moral Words	−0.013255	0.054699	0.986832	0.973578	1.000267
Authentication Type × Emotional Words	0.018812	0.006967	1.018990	1.005161	1.033009
Authentication Type × Moral-Emotional Words	−0.051778	0.000011	0.949540	0.927834	0.971753

Note: All language variables and followers are mean-centered. User type and media type use effects coding. *n* = 97,731.

**Table 3:** Negative binomial regression (DV: reposts)—GBV. Emotional language is positively associated with reposts (IRR = 1.12), whereas moral (IRR = 0.95) and moral-emotional language (IRR = 0.84) are negative predictors. The authentication main effect is negative (IRR = 0.69). Interactions are modest: authentication slightly attenuates the emotional effect (IRR = 0.98), is non-significant with moral words, and shifts the moral-emotional effect toward zero (IRR = 1.07).

	Coefficient	*p* > |z|	IRR	IRR_CI_2.5%	IRR_CI_97.5%
Intercept	−0.853224	0.000000	0.426039	0.408699	0.444116
Moral Words	−0.049441	0.000000	0.951761	0.938478	0.965232
Emotional Words	0.111466	0.000000	1.117916	1.099604	1.136533
Moral-Emotional Words	−0.169417	0.000000	0.844157	0.828182	0.860439
Log-Followers	0.477302	0.000000	1.611720	1.588509	1.635269
Authentication Type	−0.370692	0.000000	0.690257	0.652124	0.730619
Media Type	1.421567	0.000000	4.143608	3.986287	4.307139
Authentication Type × Moral Words	−0.001102	0.876981	0.998898	0.985056	1.012936
Authentication Type × Emotional Words	−0.022161	0.007877	0.978083	0.962226	0.994201
Authentication Type × Moral-Emotional Words	0.069734	0.000000	1.072223	1.051976	1.092860

Note: All language variables and followers are mean-centered. User type and media type use effects coding. *n* = 68,411.

**Table 4:** Distribution of posts per author across topics. Single-post authors dominate in all topics (SLB = 67.75%, EG = 73.33%, GBV = 77.08%), while multi-post authors account for roughly a quarter to a third (22.92–32.25%). The multi-post tail thins quickly (e.g., “>3” falls to 5.68–11.07%), but cluster sizes are highly heterogeneous across topics (ranges: SLB 1–76; EG 1–380; GBV 1–466), underscoring potential non-independence from high-activity users.

Topic				
Number of Posts Appearing in Data Set	Street-Level Bureaucracy	Education Governance	Gender-Based Violence	Mean
1	67.75	73.33	77.08	72.72
>1	32.25	26.67	22.92	27.28
>2	17	12.99	9.75	13.25
>3	11.07	8.23	5.68	8.33
>4	7.88	5.85	3.77	5.83
>5	5.99	4.46	2.72	4.39
Range	1–76	1–380	1–466	

**Figure A1:**
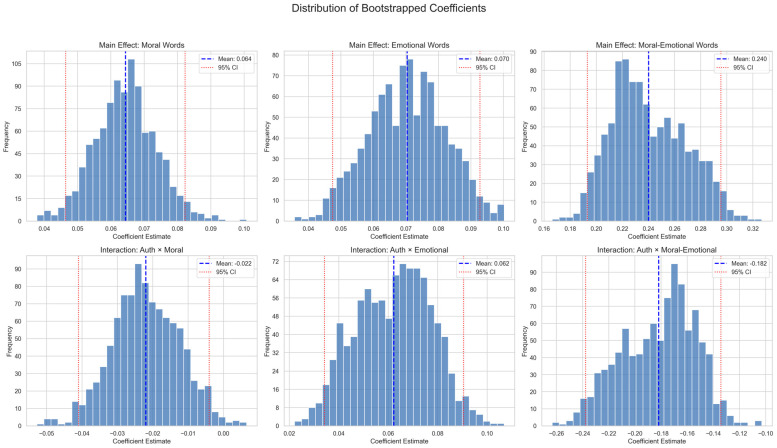
Distribution of bootstrapped coefficients for the SLB Model. The figure displays the results of 1000 cluster-aware bootstrap simulations to assess the robustness of the model’s coefficients. Each histogram shows the distribution of a coefficient’s estimate across the simulations. The dashed blue line represents the mean effect size (Mean), while the solid red lines delineate the 95% confidence interval (95% CI). A narrow confidence interval that does not cross zero indicates a robust and statistically significant effect.

**Figure A3:**
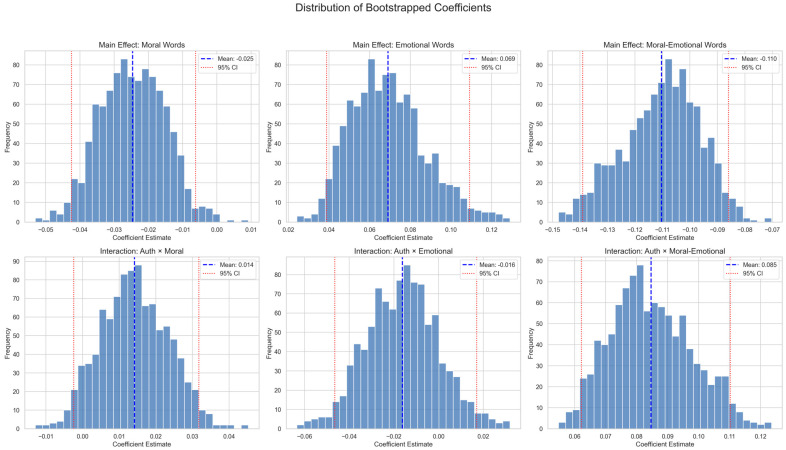
Distribution of bootstrapped coefficients for the GBV model. The figure displays the results of 1000 cluster-aware bootstrap simulations to assess the robustness of the model’s coefficients. Each histogram shows the distribution of a coefficient’s estimate across the simulations. The dashed blue line represents the mean effect size (mean), while the solid red lines delineate the 95% confidence interval (95% CI). A narrow confidence interval that does not cross zero indicates a robust and statistically significant effect.

## Data Availability

The raw data supporting the conclusions of this article will be made available by the authors on request.

## Abbreviations

The following abbreviations are used in this manuscript:

AI	Artificial Intelligence
C-MFD	Chinese Moral Foundation Dictionary
DUTIR	Information Retrieval Laboratory of Dalian University of Technology
EG	Education Governance
GBV	Gender-Based Violence
ICS	Informative Cluster Size
IRR	Incident Rate Ratio
LLMs	Large language models
MAD	Motivation–Attention–Design
ME	Moral-Emotional
NGOs	Non-Governmental Organizations
SLB	Street-Level Bureaucracy
UNHCR	the United Nations High Commissioner for Refugees
VAD	Valence, Arousal, and Dominance
WHO	World Health Organization

## Appendix A

The following robustness checks test the stability of our core predictors: the main effect of Moral-emotional words and their interaction with authentication type.

### Appendix A.1. Retaining Only Moral-Emotional Words, Its Interaction, and Controls

**Table A1:** SLB. With only moral-emotional language and controls retained, moral-emotional language strongly increases reposts (IRR = 1.456). Verification lowers baseline diffusion (IRR = 0.768) and dampens this payoff (Auth × ME IRR = 0.839). Larger audiences and media type also raise reposts (log-followers IRR = 1.79; media type IRR = 2.17).

	Coefficient	Std.Err.	*p* > |z|	Incident Rate Ratio (IRR)	IRR_CI_2.5%	IRR_CI_97.5%
Intercept	−0.217390	0.041110	0.000000	0.804616	0.742329	0.872131
Moral-Emotional Words	0.375602	0.026065	0.000000	1.455867	1.383360	1.532175
Log-Followers	0.579699	0.011191	0.000000	1.785500	1.746764	1.825095
Authentication Type	−0.263814	0.042460	0.000000	0.768116	0.706781	0.834774
Media Type	0.775174	0.040371	0.000000	2.170970	2.005811	2.349728
Authentication Type × Moral-Emotional Words	−0.175586	0.025691	0.000000	0.838966	0.797766	0.882293

**Table A2:** EG. Moral-emotional language shows a small positive association (IRR = 1.041). Verification reduces baseline diffusion (IRR = 0.773) and weakens the moral-emotional effect (Auth × ME IRR = 0.944). Followers and media type remain strong positives (IRR = 1.48 and 3.02, respectively).

	Coefficient	Std.Err.	*p* > |z|	Incident Rate Ratio (IRR)	IRR_CI_2.5%	IRR_CI_97.5%
Intercept	−0.534210	0.016998	0.000000	0.586132	0.566926	0.605989
Moral-Emotional Words	0.040000	0.011373	0.000436	1.040810	1.017867	1.064271
Log-Followers	0.389409	0.005399	0.000000	1.476108	1.460571	1.491812
Authentication Type	−0.257230	0.023808	0.000000	0.773190	0.737940	0.810125
Media Type	1.105046	0.016779	0.000000	3.019364	2.921683	3.120310
Authentication Type × Moral-Emotional Words	−0.057767	0.011364	0.000000	0.943869	0.923079	0.965128

**Table A3:** GBV. Moral-emotional language is linked to fewer reposts (IRR = 0.836). Verification lowers baseline diffusion (IRR = 0.703) but partly offsets the negative moral-emotional effect (Auth × ME IRR = 1.071). Followers and media type exert large positive effects (IRR = 1.59 and 4.28).

	Coefficient	Std.Err.	*p* > |z|	Incident Rate Ratio (IRR)	IRR_CI_2.5%	IRR_CI_97.5%
Intercept	−0.796287	0.020897	0.000000	0.451000	0.432901	0.469856
Moral-Emotional Words	−0.178625	0.009666	0.000000	0.836420	0.820723	0.852417
Log-Followers	0.465685	0.007418	0.000000	1.593105	1.570109	1.616438
Authentication Type	−0.352854	0.029390	0.000000	0.702679	0.663346	0.744345
Media Type	1.454114	0.019577	0.000000	4.280688	4.119552	4.448127
Authentication Type × Moral-Emotional Words	0.068475	0.009606	0.000000	1.070874	1.050900	1.091227

### Appendix A.2. Adding “Weibo Text Length” as a Control Variable

**Table A4:** SLB. Adding Weibo text length leaves the core results unchanged: moral, emotional, and especially moral-emotional language predict more reposts (ME IRR = 1.34). Verification lowers baseline diffusion (IRR = 0.78), dampens the moral-emotional payoff (Auth × ME IRR = 0.86), and slightly amplifies the emotional effect (Auth × Emo IRR = 1.05). Text length is negligible (*p* = 0.86).

	Coefficient	Std.Err.	*p* > |z|	Incident Rate Ratio (IRR)	IRR_CI_2.5%	IRR_CI_97.5%
Intercept	−0.266911	0.041788	0.000000	0.765741	0.705524	0.831098
Moral Words	0.065051	0.012663	0.000000	1.067213	1.041052	1.094031
Emotional Words	0.059407	0.013989	0.000022	1.061207	1.032505	1.090707
Moral-Emotional Words	0.290223	0.030306	0.000000	1.336726	1.259638	1.418532
Log-Followers	0.573758	0.011179	0.000000	1.774925	1.736459	1.814243
Weibo Text Length	0.000011	0.000060	0.858478	1.000011	0.999893	1.000129
Authentication Type	−0.248562	0.042358	0.000000	0.779921	0.717787	0.847434
Media Type	0.818020	0.041630	0.000000	2.266008	2.088458	2.458653
Authentication Type × Moral Words	−0.030702	0.012559	0.014498	0.969765	0.946185	0.993931
Authentication Type × Emotional Words	0.048822	0.013802	0.000404	1.050034	1.022009	1.078826
Authentication Type × Moral-Emotional Words	−0.149645	0.030042	0.000001	0.861014	0.811780	0.913233

**Table A5:** EG. With text length controlled, moral-emotional language shows a small positive association (IRR = 1.04), while moral and emotional language are not significant. Followers and media type remain strong positives (IRR = 1.49; 2.95). Verification reduces baseline diffusion (IRR = 0.77), weakens moral and moral-emotional effects (Auth × M IRR = 0.985; Auth × ME IRR = 0.956), and slightly amplifies the emotional effect (Auth × Emo IRR = 1.020). Text length has a tiny but significant increase (IRR = 1.00018).

	Coefficient	Std.Err.	*p* > |z|	Incident Rate Ratio (IRR)	IRR_CI_2.5%	IRR_CI_97.5%
Intercept	−0.556557	0.017126	0.000000	0.573179	0.554259	0.592745
Moral Words	0.002020	0.006677	0.762226	1.002022	0.988995	1.015221
Emotional Words	0.006198	0.006865	0.366581	1.006218	0.992770	1.019848
Moral-Emotional Words	0.035733	0.011490	0.001872	1.036379	1.013300	1.059983
Log-Followers	0.398845	0.005473	0.000000	1.490103	1.474205	1.506173
Weibo Text Length	0.000184	0.000016	0.000000	1.000184	1.000152	1.000216
Authentication Type	−0.258659	0.024131	0.000000	0.772086	0.736420	0.809480
Media Type	1.080857	0.017218	0.000000	2.947204	2.849406	3.048360
Authentication Type × Moral Words	−0.015137	0.006654	0.022906	0.984977	0.972215	0.997906
Authentication Type × Emotional Words	0.019762	0.006738	0.003358	1.019959	1.006577	1.033518
Authentication Type × Moral-Emotional Words	−0.045073	0.011486	0.000087	0.955928	0.934648	0.977691

**Table A6:** GBV. Results are robust to text length: emotional language increases reposts (IRR = 1.08), while moral and moral-emotional language decrease them (IRR = 0.95; 0.86). Verification is negative overall (IRR = 0.67) and moderates the moral-emotional penalty upward (Auth × ME IRR = 1.077); other interactions are small/NS. Followers and media type are strong positives (IRR = 1.65; 3.62), and text length has only a minute positive effect (IRR = 1.00054).

	Coefficient	Std.Err.	*p* > |z|	Incident Rate Ratio (IRR)	IRR_CI_2.5%	IRR_CI_97.5%
Intercept	−0.939071	0.021373	0.000000	0.390991	0.374951	0.407718
Moral Words	−0.053188	0.007042	0.000000	0.948202	0.935204	0.961380
Emotional Words	0.076158	0.008092	0.000000	1.079133	1.062153	1.096384
Moral-Emotional Words	−0.153362	0.009586	0.000000	0.857819	0.841853	0.874089
Log-Followers	0.498102	0.007378	0.000000	1.645596	1.621970	1.669566
Weibo Text Length	0.000541	0.000035	0.000000	1.000542	1.000473	1.000610
Authentication Type	−0.401442	0.028933	0.000000	0.669354	0.632452	0.708409
Media Type	1.287149	0.020405	0.000000	3.622444	3.480429	3.770253
Authentication Type × Moral Words	0.004250	0.006996	0.543498	1.004259	0.990583	1.018124
Authentication Type × Emotional Words	−0.010354	0.007928	0.191549	0.989699	0.974439	1.005198
Authentication Type × Moral-Emotional Words	0.074454	0.009589	0.000000	1.077296	1.057239	1.097734

### Appendix A.3. Removing the “Media Type” Control

**Table A7:** SLB. Removing media type leaves the substantive pattern intact: moral-emotional language strongly raises reposts (IRR = 1.33), moral language is modestly positive, and followers remain a major booster (IRR = 1.83). Verification lowers baseline diffusion (IRR = 0.79), dampens the ME effect (Auth × ME IRR = 0.86), and amplifies the emotional effect (Auth × Emo IRR = 1.08).

	Coefficient	Std.Err.	*p* > |z|	Incident Rate Ratio (IRR)	IRR_CI_2.5%	IRR_CI_97.5%
Intercept	0.343293	0.030056	0.000000	1.409582	1.328944	1.495113
Moral Words	0.048405	0.012447	0.000101	1.049596	1.024301	1.075515
Emotional Words	0.021084	0.013800	0.126549	1.021308	0.994055	1.049309
Moral-Emotional Words	0.285592	0.029590	0.000000	1.330549	1.255580	1.409996
Log-Followers	0.603235	0.011199	0.000000	1.828022	1.788336	1.868590
Authentication Type	−0.230176	0.042500	0.000000	0.794393	0.730903	0.863399
Authentication Type × Moral Words	−0.022805	0.012443	0.066844	0.977453	0.953904	1.001585
Authentication Type × Emotional Words	0.072603	0.013799	0.000000	1.075303	1.046610	1.104783
Authentication Type × Moral-Emotional Words	−0.150410	0.029404	0.000000	0.860355	0.812174	0.911394

**Table A8:** EG. Excluding media type shifts effects downward: moral language is only slightly positive (IRR = 1.033), while emotional and moral-emotional language are not significant. Verification remains negative (IRR = 0.85) and weakens both moral (Auth × M IRR = 0.975) and moral-emotional cues (Auth × ME IRR = 0.952). Combined with the bootstrap and threshold-slices checks, this suggests that earlier positives were partly driven by media-form mix and high-activity (often verified) users.

	Coefficient	Std.Err.	*p* > |z|	Incident Rate Ratio (IRR)	IRR_CI_2.5%	IRR_CI_97.5%
Intercept	0.136150	0.016189	0.000000	1.145854	1.110066	1.182795
Moral Words	0.032359	0.007630	0.000022	1.032888	1.017556	1.048451
Emotional Words	−0.012159	0.007287	0.095204	0.987915	0.973905	1.002126
Moral-Emotional Words	0.008760	0.013338	0.511351	1.008798	0.982767	1.035518
Log-Followers	0.386775	0.005835	0.000000	1.472225	1.455484	1.489158
Authentication Type	−0.162665	0.026295	0.000000	0.849876	0.807185	0.894825
Authentication Type × Moral Words	−0.025404	0.007637	0.000879	0.974916	0.960432	0.989618
Authentication Type × Emotional Words	0.004536	0.007291	0.533890	1.004546	0.990292	1.019005
Authentication Type × Moral-Emotional Words	−0.049634	0.013338	0.000198	0.951578	0.927025	0.976781

**Table A9:** GBV. Dropping media type sharpens contrasts: emotional language shows a stronger positive association (IRR = 1.195), while moral and moral-emotional language are more negative (IRR = 0.90; 0.76). Verification reduces baseline diffusion (IRR = 0.64) but mitigates the ME penalty (Auth × ME IRR = 1.166). Followers remain strongly positive (IRR = 1.69).

	Coefficient	Std.Err.	*p* > |z|	Incident Rate Ratio (IRR)	IRR_CI_2.5%	IRR_CI_97.5%
Intercept	−0.285021	0.021238	0.000000	0.751998	0.721338	0.783961
Moral Words	−0.103117	0.007617	0.000000	0.902022	0.888656	0.915588
Emotional Words	0.178041	0.010284	0.000000	1.194874	1.171031	1.219203
Moral-Emotional Words	−0.273849	0.009372	0.000000	0.760447	0.746605	0.774545
Log-Followers	0.525339	0.007392	0.000000	1.691032	1.666709	1.715710
Authentication Type	−0.447767	0.028629	0.000000	0.639054	0.604183	0.675937
Authentication Type × Moral Words	−0.001112	0.007614	0.883846	0.998888	0.984093	1.013906
Authentication Type × Emotional Words	−0.019505	0.010102	0.053502	0.980684	0.961458	1.000294
Authentication Type × Moral-Emotional Words	0.153685	0.009328	0.000000	1.166123	1.144997	1.187639

### Appendix A.4. Retaining Only the Interaction Involving Moral-Emotional Words

**Table A10:** SLB. Keeping only the interaction with moral-emotional (ME) language, ME remains a strong positive predictor of reposts (IRR = 1.33). Verification lowers baseline diffusion (IRR = 0.78) and attenuates the ME effect (Auth × ME IRR = 0.87). Moral and emotional language are modestly positive; followers and media are strong positives.

	Coefficient	Std.Err.	*p* > |z|	Incident Rate Ratio (IRR)	IRR_CI_2.5%	IRR_CI_97.5%
Intercept	−0.264842	0.041446	0.000000	0.767327	0.707460	0.832261
Moral Words	0.057704	0.011536	0.000001	1.059401	1.035717	1.083627
Emotional Words	0.067855	0.013628	0.000001	1.070210	1.042003	1.099179
Moral-Emotional Words	0.286592	0.028211	0.000000	1.331881	1.260238	1.407597
Log-Followers	0.572715	0.011216	0.000000	1.773075	1.734522	1.812485
Authentication Type	−0.254347	0.042549	0.000000	0.775422	0.713380	0.842861
Media Type	0.828858	0.041189	0.000000	2.290701	2.113042	2.483297
Authentication Type × Moral-Emotional Words	−0.143663	0.025637	0.000000	0.866180	0.823732	0.910815

**Table A11:** EG. With only the ME interaction retained, effects are small: emotional (IRR = 1.02) and ME language (IRR = 1.03) show slight positives, while moral is non-significant. Verification reduces baseline diffusion (IRR = 0.77) and weakens the ME effect (Auth × ME IRR = 0.95). Followers and media remain strong positives.

	Coefficient	Std.Err.	*p* > |z|	Incident Rate Ratio (IRR)	IRR_CI_2.5%	IRR_CI_97.5%
Intercept	−0.537638	0.017030	0.000000	0.584126	0.564952	0.603952
Moral Words	0.007749	0.006880	0.260018	1.007779	0.994281	1.021461
Emotional Words	0.021775	0.006948	0.001725	1.022014	1.008190	1.036027
Moral-Emotional Words	0.033407	0.011707	0.004324	1.033971	1.010516	1.057971
Log-Followers	0.389684	0.005397	0.000000	1.476515	1.460980	1.492215
Authentication Type	−0.257130	0.023826	0.000000	0.773268	0.737988	0.810234
Media Type	1.110094	0.016959	0.000000	3.034642	2.935434	3.137203
Authentication Type × Moral-Emotional Words	−0.054054	0.011350	0.000002	0.947381	0.926539	0.968691

**Table A12:** GBV. Results are polarized: emotional language increases reposts (IRR = 1.11), whereas moral and ME language reduce them (IRR = 0.95; 0.84). Verification is negative overall (IRR = 0.69) but partly offsets the ME penalty (Auth × ME IRR = 1.07). Followers and media exert large positive effects.

	Coefficient	Std.Err.	*p* > |z|	Incident Rate Ratio (IRR)	IRR_CI_2.5%	IRR_CI_97.5%
Intercept	−0.848220	0.021064	0.000000	0.428176	0.410860	0.446223
Moral Words	−0.049636	0.007076	0.000000	0.951575	0.938470	0.964864
Emotional Words	0.108148	0.008337	0.000000	1.114212	1.096154	1.132568
Moral-Emotional Words	−0.170564	0.009693	0.000000	0.843189	0.827321	0.859361
Log-Followers	0.476161	0.007393	0.000000	1.609882	1.586722	1.633381
Authentication Type	−0.371823	0.029063	0.000000	0.689476	0.651300	0.729891
Media Type	1.420245	0.019737	0.000000	4.138136	3.981114	4.301350
Authentication Type × Moral-Emotional Words	0.072037	0.009671	0.000000	1.074695	1.054517	1.095259

### Appendix A.5. Cluster-Robust Bootstrapping

To account for the non-independence of observations from users with multiple posts, we performed a cluster-robust bootstrap analysis. The procedure involved randomly sampling one post from each multi-post user and repeating this process 1000 times to generate a distribution of effect sizes. The resulting 95% confidence intervals (CIs) confirmed that the estimates for our key language variables and their interaction terms are stable, indicating the findings are not an artifact of user-level clustering.

## Appendix B

### Appendix B.1. Effect of Emotional Polarity on Repost Rates

**Table A13:** SLB. Negative emotion is associated with more reposts (IRR = 1.028, *p* < 0.001), while positive emotion is not. Both positive and negative moral-emotional scores increase diffusion (IRR = 1.084 and 1.055). Moral language is modestly positive (IRR = 1.079). Followers and media strongly raise reposts; authentication is negative.

	Coefficient	Std.Err.	*p* > |z|	Incident Rate Ratio (IRR)	IRR_CI_2.5%	IRR_CI_97.5%
Intercept	−0.207792	0.041573	0.000001	0.812376	0.748807	0.881341
Moral Words	0.075579	0.011330	0.000000	1.078509	1.054824	1.102726
Positive Emotional Score	0.004156	0.005098	0.414945	1.004165	0.994181	1.014250
Negative Emotional Score	0.027638	0.005008	0.000000	1.028024	1.017982	1.038164
Positive Moral-Emotional Score	0.080523	0.010645	0.000000	1.083854	1.061475	1.106705
Negative Moral-Emotional Score	0.053824	0.010204	0.000000	1.055298	1.034403	1.076616
Log-Followers	0.568665	0.011244	0.000000	1.765908	1.727416	1.805259
Authentication Type	−0.273876	0.044214	0.000000	0.760426	0.697304	0.829263
Media Type	0.815004	0.041677	0.000000	2.259186	2.081979	2.451475

**Table A14:** EG. Only negative emotion shows a small positive association (IRR = 1.030). Positive emotion and positive moral-emotional are not significant, whereas negative moral-emotional slightly reduces reposts (IRR = 0.988). Followers and media are strong positives; authentication is negative.

	Coefficient	Std.Err.	*p* > |z|	Incident Rate Ratio (IRR)	IRR_CI_2.5%	IRR_CI_97.5%
Intercept	−0.549141	0.016991	0.000000	0.577446	0.558532	0.597000
Moral Words	0.008885	0.006688	0.184020	1.008924	0.995786	1.022236
Positive Emotional Score	0.003300	0.002657	0.214267	1.003306	0.998094	1.008545
Negative Emotional Score	0.029804	0.003122	0.000000	1.030252	1.023968	1.036575
Positive Moral-Emotional Score	0.002167	0.005511	0.694139	1.002170	0.991403	1.013053
Negative Moral-Emotional Score	−0.011641	0.002945	0.000077	0.988426	0.982737	0.994149
Log-Followers	0.385857	0.005415	0.000000	1.470874	1.455345	1.486569
Authentication Type	−0.235552	0.023961	0.000000	0.790134	0.753885	0.828126
Media Type	1.109948	0.016852	0.000000	3.034202	2.935619	3.136095

**Table A15:** GBV. Positive emotion boosts reposts (IRR = 1.041); negative emotion is not significant. Negative moral-emotional score tilt negative (negative ME IRR = 0.993). Moral language lowers reposts (IRR = 0.959). Followers and media remain strong positives; authentication is negative.

	Coefficient	Std.Err.	*p* > |z|	Incident Rate Ratio (IRR)	IRR_CI_2.5%	IRR_CI_97.5%
Intercept	−0.730555	0.020130	0.000000	0.481641	0.463009	0.501024
Moral Words	−0.041851	0.007185	0.000000	0.959013	0.945603	0.972614
Positive Emotional Score	0.040241	0.004393	0.000000	1.041062	1.032137	1.050065
Negative Emotional Score	0.003724	0.002653	0.160375	1.003731	0.998526	1.008964
Positive Moral-Emotional Score	−0.007633	0.006387	0.232051	0.992396	0.980051	1.004897
Negative Moral-Emotional Score	−0.006962	0.003419	0.041730	0.993062	0.986429	0.999739
Log-Followers	0.458547	0.007319	0.000000	1.581775	1.559246	1.604628
Authentication Type	−0.354262	0.029656	0.000000	0.701691	0.662068	0.743686
Media Type	1.484741	0.019711	0.000000	4.413823	4.246556	4.587677

### Appendix B.2. Effects of Discrete Moral-Emotional Categories on Reposts

**Table A16:** SLB. Among moral-emotional (ME) subdimensions, only ME_Good shows a small positive association with reposts (IRR = 1.022); ME_Joy, ME_Sadness, ME_Fear, and ME_Disgust are not significant.

	Coefficient	Std.Err.	*p* > |z|	Incident Rate Ratio (IRR)	IRR_CI_2.5%	IRR_CI_97.5%
Intercept	2.198364	0.031632	0.000000	9.010261	8.468609	9.586558
ME_Joy Score per	0.152332	0.099538	0.125920	1.164546	0.958143	1.415413
ME_Good Score	0.021793	0.010856	0.044692	1.022032	1.000517	1.044011
ME_Sadness Score	0.052037	0.046180	0.259808	1.053415	0.962257	1.153209
ME_Fear Score	0.184603	0.149119	0.215731	1.202741	0.897929	1.611024
ME_Disgust Score	−0.009094	0.013691	0.506525	0.990947	0.964710	1.017897

Note: The anger and surprise categories were omitted from the model due to insufficient data for reliable estimation.

**Table A17:** EG. The ME subdimensions are mostly null; only ME_Disgust exhibits a small negative effect (IRR = 0.988).

	Coefficient	Std.Err.	*p* > |z|	Incident Rate Ratio (IRR)	IRR_CI_2.5%	IRR_CI_97.5%
Intercept	1.320753	0.018629	0.000000	3.746242	3.611925	3.885555
ME_Joy Score	0.017168	0.016244	0.290554	1.017317	0.985438	1.050226
ME_Good Score	−0.002324	0.008210	0.777152	0.997679	0.981754	1.013863
ME_Sadness Score	0.028687	0.044693	0.520958	1.029103	0.942792	1.123315
ME_Fear Score	0.118506	0.092438	0.199841	1.125814	0.939254	1.349428
ME_Disgust Score	−0.011865	0.003992	0.002956	0.988205	0.980503	0.995967

Note: The anger and surprise categories were omitted from the model due to insufficient data for reliable estimation.

**Table A18:** GBV. ME_Fear strongly promotes diffusion (IRR = 1.314), and ME_Good is also positive (IRR = 1.069). ME_Sadness is marginally negative (IRR = 0.95, *p* = 0.093); ME_Joy and ME_Disgust are not significant.

	Coefficient	Std.Err.	*p* > |z|	Incident Rate Ratio (IRR)	IRR_CI_2.5%	IRR_CI_97.5%
Intercept	1.425181	0.024268	0.000000	4.158611	3.965439	4.361192
ME_Joy Score per	0.021623	0.047039	0.645747	1.021858	0.931861	1.120547
ME_Good Score	0.066351	0.012561	0.000000	1.068602	1.042616	1.095236
ME_Sadness Score	−0.050929	0.030307	0.092870	0.950346	0.895539	1.008507
ME_Fear Score	0.272934	0.061817	0.000010	1.313814	1.163899	1.483038
ME_Disgust Score	0.001301	0.004676	0.780798	1.001302	0.992168	1.010520

Note: The anger and surprise categories were omitted from the model due to insufficient data for reliable estimation.

## Appendix C

### Appendix C.1. Human–AI Coding Run Log

**Table A19:** Environment. All human–AI coding runs were conducted in UTC+8 using Google Gemini 2.5 Pro (chat interface) on 14 July 2025 with default deterministic settings. The model used Prompt v1.0 (see Appendix C.2). The dataset comprised 349 Weibo hot-event titles from the Micro Hotspot Big Data Research Institute, where posts were pre-clustered into events and assigned unique IDs.

Time zone	UTC+8
Provider/Model	Google, Gemini 2.5 Pro (chat interface)
Access date	14 July 2025
Deterministic settings	Default
Prompt	Prompt v1.0 (see Appendix C.2)
Data	Micro Hotspot Big Data Research Institute, Weibo hot events 2024 (*n* = 349 events; provider clustered posts to events and assigned unique IDs).

### Appendix C.2. Prompt v1.0

Now we are going to do human-AI collaborative coding. The coding task is to classify 349 hot search event titles on Weibo. They need to be divided into four categories: gender-based violence (GBV), street-level bureaucrats, education governance, and others. That is to say, all events except the first three topics are classified as others, and no detailed judgment is made. Please read the definitions of the first three topics carefully and classify the events. First, you will be given a subset of events to judge. These events are random. Each event may be one of the four categories of topics. They have been manually coded. After you finish coding, you will compare the accuracy with the manual coding results. Now please read the definition: Gender-based: violence violence, discrimination or oppression against individuals, especially women and gender minorities, based on gender differences, gender identity or social gender roles. Its forms include but are not limited to physical violence, sexual violence, verbal insults, sexual harassment, humiliating remarks, online rumors, institutional injustice, etc. Such behavior is often accompanied by Patriarchal structures, gender stereotypes, and unequal power relations, and causes physical and mental harm or social exclusion to victims in the public or private sphere. Street bureaucracy: events involving frontline administrative law enforcement or public service personnel (such as urban management, traffic police, community grid workers, etc.) exercising administrative discretion, implementing policies or providing services when directly facing the public. Its core characteristics include: frontline personnel interact with the public face-to-face as policy implementers; under limited resources, vague norms or implementation pressure, individual behavior to a certain extent reflects the actual implementation of policies; events often involve the use of power, law enforcement standards, service attitudes, execution efficiency, public reaction and other content. All social conflicts, disputes, innovations or daily practices related to “how grassroots frontline public officials enforce the law, how to provide services, whether they abuse their power or neglect their duties” can be classified as “street bureaucracy” issues. Education governance: social concerns and disputes caused by rule-making, policy implementation, power and responsibility allocation and governance structure arrangements in the education system. It involves coordination and conflict between multiple This issue focuses on the performance of education policies and practices in terms of fairness, transparency, and institutional rationality, and is an important entry point for understanding the effectiveness of the education system, public trust, and social response. The events covered in the education governance topic should reflect a problem or adverse outcome caused by the structural mechanism of education.

After understanding, please make a judgment on the following events: just give the judgment result (gender-based violence (GBV), street-level bureaucrats, education governance, other) and reasons for each event.
